# Autoimmune hepatitis-specific antibodies against soluble liver antigen and liver cytosol type 1 in patients with chronic viral hepatitis

**DOI:** 10.1186/1740-2557-4-2

**Published:** 2007-02-04

**Authors:** Eirini I Rigopoulou, Maria Mytilinaiou, Ourania Romanidou, Christos Liaskos, George N Dalekos

**Affiliations:** 1 Department of Medicine, Academic Liver Unit and Research Laboratory of Internal Medicine, Larissa Medical School, University of Thessaly, Larissa, Greece; 2 Institute of Biomedical Technology (BIOMED), Center for Research and Technology-Thessaly (CE.RE.TE.TH), Larissa, Greece

## Abstract

**Background:**

Non-organ specific autoantibodies are highly prevalent in patients with chronic hepatitis C (HCV). Among them, anti-liver kidney microsomal type 1 (LKM1) antibody – the serological marker of type 2 autoimmune hepatitis (AIH-2)- is detected in up to 11% of the HCV-infected subjects. On the other hand, anti-liver cytosol type 1 antibodies (anti-LC1) – either in association with anti-LKM1, or in isolation- and anti-soluble liver antigen antibodies (anti-SLA) have been considered as useful and specific diagnostic markers for AIH. However, their specificity for AIH has been questioned by some recent studies, which have shown the detection of anti-LC1 and anti-SLA by immunoprecipitation assays in HCV patients irrespective of their anti-LKM1 status. The aim of the present study was to test the anti-LC1 and anti-SLA presence by specific enzyme linked immunosorbent assays (ELISAs), in a large group of Greek HCV-infected patients with or without anti-LKM1 reactivity as firstly, immunoprecipitation assays are limited to few specialized laboratories worldwide and cannot be used routinely and secondly, to assess whether application of such tests has any relevance in the context of patients with viral hepatitis since antibody detection based on such ELISAs has not been described in detail in large groups of HCV patients.

**Methods:**

One hundred and thirty eight consecutive HCV patients (120 anti-LKM1 negative and 18 anti-LKM1 positive) were investigated for the presence of anti-LC1 and anti-SLA by commercial ELISAs. A similar number (120) of chronic hepatitis B virus (HBV) infected patients seronegative for anti-LKM1 was also tested as pathological controls.

**Results:**

Six out of 18 (33%) anti-LKM^pos^/HCV^pos ^patients tested positive for anti-LC1 compared to 1/120 (0.83%) anti-LKM^neg^/HCV^pos ^patients and 0/120 (0%) of the anti-LKM1^neg^/HBV^pos ^patients (p < 0.001 for both comparisons). Anti-SLA antibodies were not present in any of the HCV (with or without anti-LKM1) or HBV-infected patients.

**Conclusion:**

We showed that anti-LC1 and anti-SLA autoantibodies are not detected by conventional assays in a large group of anti-LKM1 negative patients with chronic hepatitis B and C infections. Based on these results we cannot find any justification for the application of anti-LC1 and anti-SLA tests in the routine laboratory testing of viral hepatitis-related autoantibody serology with the only potential exception being the anti-LC1 screening in anti-LKM1^pos^/HCV^pos ^patients.

## Background

Non-organ specific autoantibodies (NOSA), particularly smooth muscle antibodies (SMA) and antinuclear (ANA) antibodies are highly prevalent in patients with chronic hepatitis C virus (HCV) infection [1-7]. Anti-liver kidney microsomal type 1 (LKM1) antibody – the serological marker of type 2 autoimmune hepatitis (AIH-2)- is also detected in up to 11% of the HCV-infected subjects [1,8-11]. Anti-liver cytosol type 1 (LC1) antibodies have originally been described either in association with anti-LKM1, or in isolation, and in both instances define a clinical entity indistinguishable from AIH-2 [12,13]. Anti-LC1 has also been found occasionally in anti-LKM1 positive chronic hepatitis C virus (HCV) infected patients [10,14]. Detection of anti-soluble liver antigen antibodies (anti-SLA) was initially considered to identify a third type of AIH seronegative for the conventional ANA, SMA, anti-LKM1 autoantibodies [15] but recent studies indicate that it can also be present in conjunction with other AIH-specific antibodies suggesting that anti-SLA is rather an additional important marker for the diagnosis of type 1 AIH, than a marker of a third type of AIH [11,16-19].

Hence, anti-LC1 and anti-SLA autoantibodies appear useful diagnostic markers for AIH but their accurate detection was until recently hampered by the fact that anti-LC1 is obscured by the concurrent presence of anti-LKM1 using the indirect immunofluorescence (IIFL) routine screening, while anti-SLA are undetectable by IIFL [9-11]. In recent years, the molecular targets of anti-LC1 and anti-SLA have been identified as formiminotransferase cyclodeaminase (FTCD) and UGA tRNA suppressor associated antigenic protein (tRNP^(Ser)Sec^), respectively and commercial enzyme linked immunosorbent assay (ELISA) kits for their detection have become available [9,20-25]. Their specificity for AIH, however, has been questioned by studies from the group of Alvarez [26,27]. By immunoprecipitation of radiolabeled human FTCD, Beland et al have found that anti-FTCD antibodies are present in 10% of anti-LKM1 positive and 15% of anti-LKM1 negative chronic HCV infected patients [26]. Of interest and using a similar approach, the same group has detected anti-SLA antibodies in 28% of anti-LKM1 positive and in 12% of anti-LKM1 negative HCV infected patients [27]. These findings challenge the prevailing notion that antibodies against human FTCD and tRNP^(Ser)Sec ^are highly specific for autoimmune liver diseases [20-25,28,29]. According to the authors of the abovementioned studies [26,27], anti-LC1 and anti-SLA autoantibodies can be regarded as serological markers of autoimmunity and need to be tested when investigating autoimmunity, especially in chronic HCV infection.

Immunoprecipitation assays, however, are limited to few specialized laboratories worldwide and cannot be used routinely. Indeed, most of the non-specialized laboratories have started making use of kit-based semi-automated commercial anti-LC1 and anti-SLA ELISAs. The question as to whether application of such tests has any relevance in the context of patients with viral hepatitis still remains unanswered, as antibody detection based on such ELISAs has not been described in detail in large groups of patients with chronic viral hepatitis B and C.

The aim of the present study was to test anti-FTCD (anti-LC1) and anti-tRNP^(Ser)Sec ^(anti-SLA) antibodies, in a large number of Greek HCV-infected patients with or without anti-LKM1 seropositivity. A similar number of chronic hepatitis B virus (HBV) infected patients was also tested as pathological controls. We were able to show that there is no reason for additional routine laboratory testing for anti-LC1 and anti-SLA antibodies in HCV-infected patients unless there are anti-LKM1 antibodies present.

## Methods

### Patients

One hundred and twenty consecutive patients with chronic hepatitis C (mean age 47 ± 12.3 standard deviation (SD) years; 62 female), seronegative for anti-LKM1 (anti-LKM^neg^/HCV^pos^) by IIFL followed at the Department of Medicine, Academic Liver Unit, University of Thessaly Medical School were studied. The diagnosis of chronic HCV infection was based on: (a) detection of anti-HCV antibodies using a third-generation ELISA (Murex Diagnostics, Temple Hill, Dartford, UK) at least twice within 6 months before their enrolment into the study; and (b) active virus replication as defined by the detection of HCV RNA using a commercially available qualitative PCR kit (HCV Monitoring Cobas Amplicor, Roche, Geneva, Switzerland), as described previously [1,2,30]. At the time of serum sample collection, 45 patients had completed antiviral treatment with alpha-interferon (IFN-a) alone or in combination with ribavirin. All patients were negative for the following viral markers tested using commercial kits (Abbott Diagnostic Kits, North Chicago, IL, USA): Hepatitis B surface Antigen (HBsAg), anti-HBsAg antibody (anti-HBs), anti-Hepatitis B core antibody (anti-HBc), and anti-human immunodeficiency virus (anti-HIV, VIDAS HIVDUO Bio MER^©^IEUX, Marcy-l'Etoile, France). Histological, serological and clinical details of these patients have been described in previous reports [1,2,30]. The results obtained by ELISA testing of the anti-LKM1 negative patients with hepatitis C were compared to those obtained by testing of 18 anti-LKM1^pos^/HCV^pos ^patients (mean age 47 ± 11.3 years; 12 female) and 120 anti-LKM1^neg^/HBV^pos ^Greek patients (mean age 49.1 ± 11.2; 81 male) all HBV-DNA positive by a sensitive PCR kit (HBV Monitor Cobas Amplicor, Roche; cut-off: 200 copies/ml) and negative for HCV markers.

### Autoantibody detection

Presence of ANA, SMA, anti-LKM1, anti-LC1 and anti-mitochondrial antibodies was initially detected by IIFL on 5 μm frozen sections of in-house rodent multi-organ (kidney, liver and stomach) tissue substrates using as revealing reagent an anti-total human IgG fluorescein isothiocyanate conjugate (Dako Ltd, High Wycombe, Bucks, UK), as previously described [1,9]. Briefly, diluted sera (1/40) in phosphate buffered saline (PBS) were tested on in-house snap-frozen sections of rat liver, kidney and stomach. Positive sera were titred by double dilution to extinction. The patterns of reactivity were assessed under a fluorescence microscope (Orthoplan, Leitz, Wetzlar, Germany). Anti-LKM1, anti-LC1 and anti-SLA reactivity was also evaluated by Western immunoblotting using both human microsomal and cytosolic fraction as antigen sources (Euroimmun, Lübeck, Germany). Commercially available ELISA (Euroimmun) were used for the detection of anti-FTCD (anti-LC1) and tRNP^(Ser)Sec ^(anti-SLA) autoantibodies, according to the manufacturer's instructions. Antibody binding titres expressed as optical density (OD) ^testserum^/OD ^calibrator ^and extinction values of serum samples exceeding those of the calibrator (OD ^testserum^/OD ^calibrator ^> 1) were considered positive.

### Inhibition studies

To investigate whether the 58–60 kDa band immunofixed by anti-LC1 is FTCD, inhibition experiments were performed using the anti-LC1 positive serum, diluted at 1/200, and pre-incubated with solid phase recombinant FTCD (Euroimmun), as previously described [31,32].

Each patient gave informed consent to participate in this study. The Local Ethical Committee of The Medical School, University of Thessaly approved the study protocol.

### Statistical analysis

Data are presented as percentages (%) or mean ± SD unless otherwise stated. The differences between different groups were compared using t-test, the Mann Whitney U test, χ^2^, (two by two with Yates' correction) and Fisher's exact test as appropriate. Two-sided *p *values < 0.05 were considered as statistically significant.

## Results and discussion

**Figure 1:**
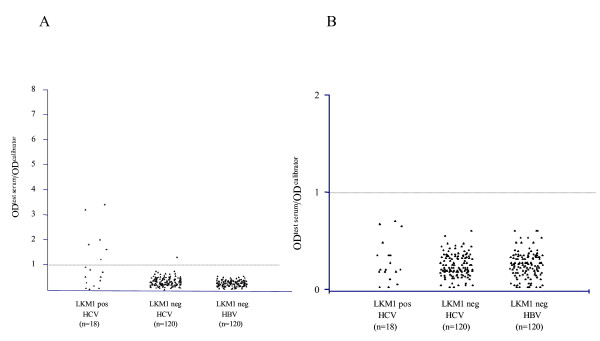
(A). ELISA titers of anti-formimino-transferase cyclodeaminase (anti-FTCD) antibodies, known also as anti-liver cytosolic type 1 antibodies (anti-LC1) and (B). titers of anti-UGA tRNA suppressor associated antigenic protein (tRNP^(Ser)Sec^) antibodies, known also as anti-soluble liver antigen antibodies (anti-SLA) in 18 liver kidney microsomal type 1 (LKM1) positive chronic hepatitis C virus (HCV) infected patients; 120 LKM1 negative chronic HCV infected patients; and 120 LKM1 negative hepatitis B virus (HBV) infected patients. According to the manufacturer's instructions, autoantibody reactivity is considered positive when optical density (OD)^test serum^/OD^calibrator ^> 1.

**Figure 2:**
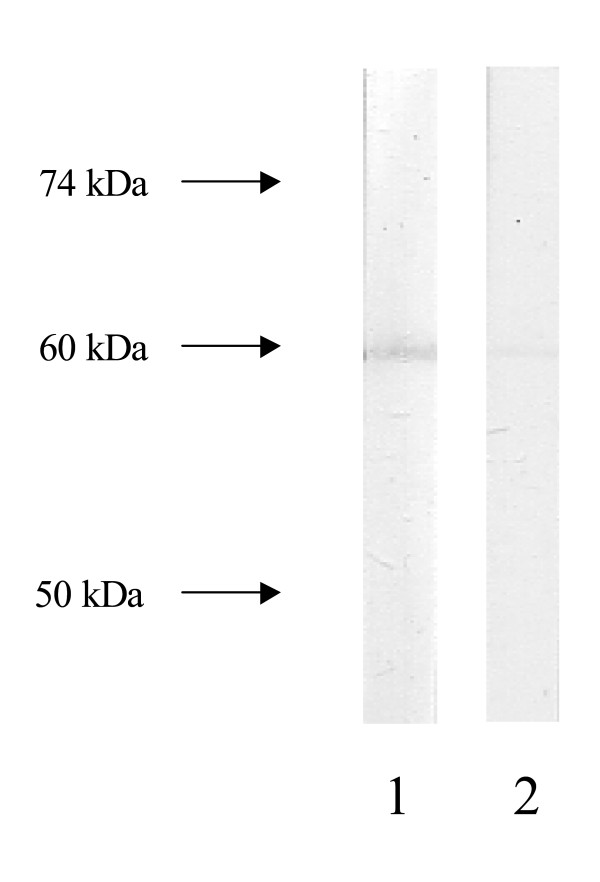
Immunoblot pattern produced by a serum sample from a liver kidney microsomal type 1 (LKM1) negative chronic hepatitis C virus (HCV) infected patient with anti-formimino-transferase cyclodeaminase (FTCD) antibodies on electrophoretically separated human liver homogenate (lane 1). The 58–60 kDa immunofixed band almost completely abolished when the reactive serum was pre-incubated with recombinant FTCD as solid phase competitor (lane 2).

Prevalence of various NOSA is summarised in Table 1. Anti-FTCD (anti-LC1) antibodies were present in 6/18 (33%) anti-LKM^pos^/HCV^pos ^compared to 1/120 (0.83%) anti-LKM^neg^/HCV^pos ^patients (a 47-years old male patient at the third month of IFN-a treatment with no evidence of aminotransferases flare and without history of extrahepatic immunopathological manifestations) and 0/120 (0%) of the anti-LKM1^neg^/HBV^pos ^patients (p < 0.001 for both; Figure 1A). By western blot using a human liver cytosolic fraction as substrate, the anti-FTCD positive anti-LKM^neg^/HCV^pos ^case immunofixed a relatively weak 58–60 kDa band corresponding to LC1 autoantigen (Figure 2, lane 1). This band was almost completely abolished when the reactive serum was pre-incubated with recombinant FTCD as solid phase competitor (Figure 2, lane 2) indicating that is due to anti-FTCD reactivity. Repeat IIFL of this case revealed absence of a pattern typical of anti-LC1; there was no staining of cytoplasm of liver cells with relative sparing of the centrilobular area, even when the serum was tested at dilution of 1/20.

**Table 1:** Prevalence of non-organ specific autoantibodies detected by indirect immunofluorescence (IIFL) or enzyme linked immunosorbent assay (ELISA) in patients with chronic hepatitis C and B.

	Anti-LKM1 (IIFL)	Anti-LC1 (ELISA)	Anti-SLA (ELISA)	ANA (IIFL)	SMA (IIFL)
LKM1^pos^/HCV^pos^	18/18 (100%)*	6/18 (33%)	0/18 (0%)	5/18 (27.7%)**	6/18 (33%)***
LKM1^neg^/HCV^pos^	0/120 (0%)	1/120 (0.8%)	0/120 (0%)	45/120 (37.5%)**	61/120 (50.8%)***
LKM1^neg^/HBV^pos^	0/120 (0%)	0/120 (0%)	0/120 (0%)	23/120 (19.2%)**	15/120 (12.5%)***

LKM1, liver kidney microsomal type 1; LC1, liver cytosol type 1; SLA, soluble liver antigen; ANA, anti-nuclear antibody; SMA, smooth muscle antibody; pos, positive; neg, negative; *Confirmed by Western immunoblotting (see Methods section); **Low titers of ANA were detected in the three groups (mean titer: 1/168, 1/148 and 1/140, respectively); ***Low titers of SMA were detected in the three groups (mean titer: 1/134, 1/121 and 1/119, respectively).

Anti-tRNP^(Ser)Sec ^(anti-SLA) antibodies were not present in any of the HCV (with or without anti-LKM1 reactivity) or HBV infected patients (Figure 1B). Similar data we have obtained when an anti-SLA ELISA of another manufacturer (Inova Diagnostics, San Diego, California, USA) was used (data not shown).

Chronic HCV infection is frequently characterized by an altered immune homeostasis as it has become evident by the high prevalence of NOSA, and in particular SMA and ANA which, according to some reports, exceeds 50% of the infected population [1-7]. Anti-LKM1 antibody – the serological marker of type 2 AIH- is also detected in up to 11% of the HCV-infected subjects [1,8-11]. Under this context, the detection of anti-LC1 and anti-SLA antibodies in a significant proportion of HCV patients in the two recent studies – irrespective of the anti-LKM1 status – [26,27] could not be a surprising finding as it might further support the autoimmune propensity of the virus.

The findings of the present study however, suggest that commercially available assays are unable to detect anti-LC1 and anti-SLA antibodies in a large number of anti-LKM1 negative patients either with chronic HCV or with chronic HBV infection. On the contrary, we found that a considerable proportion of anti-LKM^pos^/HCV^pos ^patients (33%) had detectable anti-LC1 antibodies by ELISA, which is in accordance with previous studies from Italy [14].

The question arises as to whether the inability of the detection of anti-LC1 and anti-SLA antibodies by ELISAs in patients with chronic HCV infection is due to the superior sensitivity of the immunoprecipitation assays used by Beland et al [26] and Vitozzi et al [27]. Radioligand assays of higher sensitivity than the conventional assays have increasingly been reported in the autoantibody diagnostic setting of a plethora of autoimmune diseases such as diabetes, multiple sclerosis, systemic lupus erythematosus and AIH [1,28,33-38]. There are still some reservations however, as to whether this is the sole reason for the discrepant results between ELISAs and immunoprecipitation assays specific for anti-LC1 and anti-SLA detection. First, it has been generally agreed that as equipment becomes more sophisticated, higher diagnostic sensitivity of an assay frequently comes at the expense of a lower specificity [38]. Beland et al [26] and Vitozzi et al [27] argue that this cannot be the case for their immunoprecipitation anti-LC1 and anti-SLA assays because they can detect these autoantibodies in patients with chronic hepatitis C but not in other pathological controls. These investigators, in particular, were unable to detect anti-LC1 in any of the 22 patients with type 1 AIH or the 25 patients with other unrelated chronic liver diseases (which however did not describe in detail).

The focus of the present study was not the validation of the findings of the French/Canadian group. We rather decided to investigate the prevalence of anti-LC1 and anti-SLA in anti-LKM1^neg^/HCV^pos ^patients by commercial ELISAs and to test their diagnostic utility in the day-to-day clinical practice. Our assumption has been based on the fact that if anti-LC1 and anti-SLA are present in anti-LKM1^neg^/HCV^pos ^patients and these antibodies can be detected by routinely performed ELISAs, clinicians should be aware and request for their detection. We have found that neither anti-LC1 nor anti-SLA autoantibodies are detectable in anti-LKM1 negative patients with chronic viral hepatitis C or B by ELISAs.

## Conclusion

We showed that anti-LC1 and anti-SLA autoantibodies are not detected by conventional assays in the vast majority of patients with chronic viral hepatitis B and C. Based on these results we cannot find any justification for the application of anti-LC1 and anti-SLA tests in the routine laboratory testing of viral hepatitis-related autoantibody serology unless anti-LKM1 antibodies are present in HCV-infected patients. In the latter case, only anti-LC1 screening could be of interest. Exchange of sera and standardization of the methodology employed, whether it is radioligand, western blot or ELISA is urgently warranted to address definitely whether anti-LC1 and anti-SLA autoantibodies are serological markers of chronic hepatitis C.

## Abbreviations

AIH, autoimmune hepatitis; SMA, smooth muscle antibody; ANA, antinuclear antibody; anti-LKM1, liver kidney microsomal type 1 antibody; IIFL, indirect immunofluorescence; anti-LC1, liver cytosol type 1 antibody; anti-SLA, antibody against soluble liver antigen; HCV, hepatitis C virus; HBV, hepatitis B virus; FTCD, formiminotransferase cyclodeaminase; tRNP^(Ser)Sec^, UGA tRNA suppressor associated antigenic protein; ELISA, enzyme linked immunosorbent assay; IFN-a, alpha-interferon.

## Competing interests

The author(s) declare that they have no competing interests.

## Authors' contributions

EIR and GND had the original idea for the study and wrote the paper; MM, OR and CL carried out the autoantibodies serological tests as well as the inhibition experiments and along with ER performed the statistical analysis; EIR and GND contributed to the final version of the article. All authors have read and approved the final version of the manuscript.
